# Annotations

**Published:** 1888-09-29

**Authors:** 


### Annotations.

**Brag or Benevolence?**

Seldom has that pushing and elbowing vulgarity which is so conspicuous a characteristic of men and women of all ranks in the present day received a sounder buffet on the forefront than in the recently-published diary of the late Emperor Frederick of Germany. All men knew he was a man of benevolent mind, who concerned himself much with duty and little with pomp and show. Not a few, whose intelligence was quite inadequate to the understanding of a reasonable and unselfish temper, heartily despised him for his modesty. That Bismarck should have made the German Empire—Bismarck, the man of grim countenance, of words of thunder, of deeds of lightning—that he should have made the empire, the stagey braggarts could understand. That even the Emperor William, a man of fixed purpose and sure though slow intelligence, should have accomplished the making of it—that, too, was comprehensible. But that the calm and gentle Frederick, the man who was greater than the king, who, instead of being swollen and distended with pride on account of his position and power, really looked down on both position and power from the serene height of a profound and comprehensive intelligence—that he should have been the first to conceive, the first to propound, and the first to make possible and actual the unity of the German Empire—this is a revelation which may well both confound the vulgar braggarts and help the modest to preserve their faith in moral worth. In no department of life was a conspicuous example of modesty more required than in those regions which appertain to politics and high social station. Politicians in this country, as well as in others, seem to think that the only method of advancement is that of taking a running leap at the highest possible station, and then sounding a trumpet to all the world that there you are, that you are the man, and that there you intend to remain. The method of promotion for proved service is entirely out of date. The same is true of social advancement. Men and women, for women are quite as vulgar and undignified as men in this regard, cannot endure to be anywhere except at the summit of the social gooseberry bush. They buzz and flutter, and flap their wings, and sting and bite, like so many insane wasps in an insect lunatic asylum. Contemptible insects! Of no more real value than to be treated like other wasps and suffocated by a charge of schoolboys' gunpowder. The only true way to a high place in the world's esteem is that of high and worthy service for the world. What does the present generation care for the emperors, the dukes, and the fashionable dames of the fifth century, as such? Not one straw. But for the noble literature of fishermen and tax collectors, even of the first century, the world does not know how to care enough. Let every man be content to do his own peculiar work well, to devote such portion of his time and mental energy as a high sense of duty prompts to public life, and then to take such reward as prudence and a generous world will freely offer him. Stolen honours, like stolen goods, can only please him who is at heart a thief.

**"A Sweated" Lady.**

The heroine of the "sweating system" at the present moment seems to be Miss Beatrice Potter. This lady went neither to employers nor employed for information, but sought work herself in one of the tailors' shops, and stuck to it until she made herself as thoroughly familiar with the ins and outs of the system as if she had been compelled to live by it. Miss Potter, although a lady of means and position, has worked in Whitechapel, Spitalfields, and Mile End. As may be supposed, her evidence constitutes a highly-important part of that which was submitted to the Inquiry Commission, and which has since been published in a Blue Book. She and other beneficent persons got trustworthy evidence about 1,300 different sweating-shops. That there should be so many as 1,300 in a single district of London is appalling. In one little slop-shop in which Miss Potter worked the price for coatmaking ranged from 1*s.* to 2*s.* The output in the week during which she worked in the little shop was 80 coats at 1*s.* apiece. To make this number, the "sweater" had to employ two "machinists," a "general" hand, and a girl. The girl earned 2*s.* in the week, with lodging and food. The "sweater" himself did all the basting, fixing, and pressing, and worked tremendously long hours. As the result of her investigations, Miss Potter arrives at two practical conclusions, and these both economists and philanthropists should note, as indicating the lines on which the improvement of the class under consideration will have to be sought. The first point is that those who are sweated live and work under a system of "unregulated" labour. They are outside the operation of the Factory Act and they have no trade union. Much of their sufferings depend upon these circumstances. They are more or less free lances in the army of labour, and their want of combination makes them powerless for self-defence, whilst their accidental exclusion from the Factory Act leaves them without defence from the law. The second conclusion at which Miss Potter arrives is that the only way of really stopping sweating is to enforce a higher standard of living. The fact which makes the "sweating system" possible is that there are in large towns too many labourers and too little work. The country is depleted, the town is gorged. The fields are returning to desolation and savagery for want of labourers; the labourers in the towns are falling into starvation and savagery for want of work. Why will not people see that there is plenty of work for unskilled labour if it were free to cultivate the land? The highest authorities tell us, and we know they are right, that our markets are flooded with foreign produce, because home produce is not to be had; that our fields are among the best producing fields in the world, but they cannot produce for want of labour; and that labourers are starving in the streets, whilst fields are waiting to be tilled. Can anything be more terrible and more heartbreaking than this? We have universities by the dozen, Members of Parliament by the hundred, learned professors, scientific men, and book writers by the thousand, and nobody can solve the simple problem of bringing starving labourers into the empty and desolate fields. What stands in the way? Whatever it may be it is wicked and intolerable, and ought to be straightway removed by the strong hand of legislative authority.

**Committee-men and Business Contracts.**

There has been a good deal of animated discussion at Paisley concerning the propriety or otherwise of members of the Hospital Board of Management entering into contracts to supply the hospital with necessary articles of consumption and use. It may be that some member of the Paisley Board has long observed with jealous eye the nice pickings which are to be made out of hospital contracts, and has asked himself why he, too, should not have his fair and reasonable share of them. Why, indeed? He might very plausibly argue that if he had not been a hospital manager he would have been free to cater for the contracts. Why should his connexion with the institution be allowed to prejudice his private interests? Nay, more, is he not as a manager rendering the hospital signal service, and that without fee or reward; and ought he not, on the principle of fair compensation, not only to be allowed to cater as freely for contracts as all other men, but even to have some degree of preference because of the unpaid work he does for the sake of charity? This is a very plausible argument, and when it is buttressed by self-interest, may, to certain minds, be absolutely convincing and irrefutable. It is, however, like many other carefully-constructed pieces of dialectic, of no significance at all when touched with the finger of straightforward common sense. In the abstract there is no reason whatever why a hospital manager should not hold contracts for hospital supplies. In the concrete there is every reason. The beginning of the practice would be the beginning of distrust, misunderstanding, and antagonism among the members of the committee; the extensive prevalence of it would inevitably lead to jobbery and corruption of the most shameful kind. There are plenty of examples and warnings among large public bodies, which we need not name, but which are conclusive on the practical aspect of the question. Hospital managers must be, like Cæsar's wife, altogether above suspicion. A man who permits himself to be appointed to a hospital committee must accept the position with all its drawbacks and disabilities as well as with its honours and advantages. He should on no account use his position for his own private purposes, whatever they may be. At Paisley the question has been discussed with some warmth, and the feeling is so universally strong against the acceptance of contracts by members of the committee that probably no more will be heard of it in that quarter. We avail ourselves of the occasion to call the attention of other committees to a question which may at any time become urgent, which is of great practical importance, and which admits of only one solution.

